# Structural equation modeling of female gait attractiveness using gait kinematics

**DOI:** 10.1038/s41598-023-45130-2

**Published:** 2023-10-19

**Authors:** Hiroko Tanabe, Kota Yamamoto

**Affiliations:** 1 Institutes of Innovation for Future Society, Nagoya University, Furo-cho, Chikusa-ku, Nagoya-shi, Aichi, 464-8601 Japan; 2 Japan Society for the Promotion of Science, 5-3-1 Kojimachi, Chiyoda-ku, Tokyo, 102-0083 Japan; 3 Graduate School of Informatics, Nagoya University, Furo-cho, Chikusa-ku, Nagoya-shi, Aichi, 464-8601 Japan

**Keywords:** Human behaviour, Social behaviour

## Abstract

In our social lives, movement’s attractiveness greatly affects interpersonal cognition, and gait kinematics mediates walkers’ attractiveness. However, no model using gait kinematics has so far predicted gait attractiveness. Thus, this study constructed models of female gait attractiveness with gait kinematics and physique factors as explanatory variables for both barefoot and high-heel walking. First, using motion capture data from 17 women walking, including seven professional runway models, we created gait animations. We also calculated the following gait kinematics as candidate variables to explain walking’s attractiveness: four body-silhouette-related variables and six health-related variables. Then, 60 observers evaluated each gait animation’s attractiveness and femininity. We performed correlation analysis between these variables and evaluation scores to obtain explanatory variables. Structural equation modeling suggested two models for gait attractiveness, one composed of trunk and head silhouette factors and the other of physique, trunk silhouette, and health-related gait factors. The study’s results deepened our understanding of mechanisms behind nonverbal interpersonal cognition through physical movement and brought us closer to realization of artificial generation of attractive gait motions.

## Introduction

In our social lives, the body’s attractiveness and its movements or behavior play an important role in interpersonal cognition. Several studies in evolutionary psychology and zoology have reported that animals’ standard of beauty, including that of human beings, is associated with psychological processes that detect health-related features^1–6^. Regarding human women’s attractiveness, lumbar curvature^7^, which is related to reproductive function^8–11^, and waist-to-hip ratio (WHR)^6,12^, which is important for gender discrimination^13,14^, have been reported as important factors in bodily attractiveness. In contrast, in sociocultural contexts, society shapes not only our personalities and behaviors but also our physical appearance^15^, and this has given rise to elements of physical attractiveness that contradict an individual’s health. For example, mass media exert societal pressures toward underweight body appearance as a beauty icon or an embodiment of gender role norms for girls^16–20^. Along with this, women with a lower BMI^21^ and fat mass^22^ than the physiologically healthy range have been reported to be considered relatively more attractive. In addition, although wearing high heels is risky for orthopedic health^23^, many women wear them to enhance their attractiveness^24,25^. Thus, various and partially contradictory factors influence our perception of attractiveness. In fact, no statistical model has yet been constructed to explain bodily attractiveness, so we tackled the challenge of being first to construct a model that integrates multiple elements contributing to physical attractiveness.

The human gait’s kinematic characteristics affect evaluation of interpersonal attractiveness^24,26,27^ and of personal impressions, character, and emotion^28–30^. In our previous study, we examined gait dynamics from the perspective of expressed, rather than perceived, attractiveness to find that women’s biomechanical strategy for their individual gait’s attractiveness involved showcasing femininity, fertility, and youth and that such an “attractive-conscious” gait was perceived as attractive^31^. The biomechanical strategy for expressing gait attractiveness included a backward arm swing, lumbar curvature, which created a feminine silhouette emphasizing the chest, and a head silhouette established by a forward tilt and horizontal shaking. In addition, knee extension at the push-off phase, which is related to walkers’ health^32–34^, was also such a strategy. Observers evaluated attractive-conscious gait as more attractive than normal walking, suggesting that the kinematic parameters above could be explanatory variables for women’s gait attractiveness. Besides these parameters, from an evolutionary psychological viewpoint, general gait parameters associated with health or aging, such as stride CV (coefficient of variation), cadence, clearance, symmetry, and toe-off angle^35–40^, are also candidates for explanatory variables to explain gait attractiveness. In addition, from a sociocultural perspective, physique factors such as BMI, height, and weight also influence gait attractiveness. In this study, therefore, we attempted to construct a model that explains women’s gait attractiveness by using as explanatory variables gait kinematics and physique factors related to feminine bodily silhouettes and to health.

Body dynamics provide gender information, particularly when gender morphological cues are ambiguous^41,42^. Femininity in motion is believed to be embodied by multiple factors such as pelvic obliquity, arm swing, and torso rotation^43^. We can also accurately detect gender from point-light displays of walking figures: the more hip sway, the more feminine; the more shoulder translations, the more masculine^44,45^. Additionally, especially in women, gender-type characteristics, that is, femininity in morphological features and motion, could be strongly associated with attractiveness because in sociocultural norms, strength and physical ability are featured in the context of men’s sports^46^, while performance for women tends to be evaluated by aesthetic characteristics rather than physical ability, such as in ballet^47^. D’Argenio et al.^48^ revealed the relationship between body dynamics, femininity, and attractiveness: females’ body poses with less dynamism were preferred, and such dynamism led to observers’ feelings of pleasantness through femininity. Therefore, gait dynamics could also lead to attractiveness via observers’ perception of femininity. Another causal relationship could be that perception of attractiveness gives rise to femininity—a concept not examined in the study by D’Argenio et al.^48^.

As described above, the attractiveness of female gaits could be associated with various characteristics of form and movement, including physique-related factors such as BMI and WHR, lumbar curvature, and gait parameters related to health and age. According to the information processing model of neuroaesthetics^49,50^, an observer’s perception of an aesthetic object comprises visual information processing, impression evaluation, and emotional reaction to the object. Each of these processes can be further deconstructed. Once the basic visual features of the object have been received, the information is collated through higher-level cognitive and attentional processes to form impression evaluations and final decision-making regarding the object’s visual appeal. When applied to evaluating gait attractiveness, the walking motion as a stimulus is initially deconstructed into visual features (physical and kinematic), which are sorted and processed. Then, higher-level cognitive (executive processes) and attentional processes are applied to evaluate the walkers’ gait attractiveness and femininity. This study clarifies the visual features in the initial stage of this process of recognizing gait attractiveness and investigates the relationship between gait parameters and a walker’s physique as visual features that affect observers’ attractiveness evaluation. This will contribute to our understanding of the information processing involved in the evaluation of the beauty of physical movement. Furthermore, it may deepen our understanding of the neurology of such perceptions and provide a definition for physical beauty and attractiveness.

The research questions of this study were as follows: Are gait attractiveness and femininity correlated with the gait parameters related to health and/or aging and with gait parameters used to express attractiveness by the walkers themselves? If so, is it possible to use such parameters to construct models that explain the attractiveness and femininity of the female gait? To tackle these questions, we first conducted correlation analysis between each parameter and the impression evaluation scores. Our analysis was based on the following three hypotheses:

1. There is a correlation between physique factors and impression scores. Specifically, low BMI and WHR are related to gait attractiveness.
2. The gait parameters used by walkers to showcase gait attractiveness, i.e., backward arm swing, lumbar curvature, forward tilt, and horizontal shaking of the head^31^, are positively correlated with attractiveness and femininity scores.
3. The gait parameters related to health and aging, i.e., knee extension, small stride CV, large cadence, clearance, symmetry, and toe-off angle^35–40^, are positively correlated with impression ratings.

The final goal of this study was to construct models of female gait attractiveness with gait kinematics and physique factors as explanatory variables. We constructed models of both barefoot and high-heel walking, and by comparing them, we attempted to consider the meaning and purpose of women wearing high heels while taking orthopedic risks. In the models, we also examined the relationship between femininity and attractiveness by using observers’ evaluations of femininity and attractiveness in gait animations. This would clarify elements of the body and its dynamics during walking that embody attractiveness and/or femininity. The study would not only deepen our understanding of what bodily cues cause us to perceive physical attractiveness and femininity, but also allow us to create informatic values, for instance, predicting physical attractiveness and artificially generating attractive bodies and biological motions. It should be noted that our data was obtained from Japanese participants only, so our results are likely to be biased toward Japanese culture. The applicability to other locations and cultures requires further investigation.

## Methods

All the study’s procedures were conducted according to the Declaration of Helsinki and approved by Nagoya University’s Ethics Committee. All participants provided written informed consent to participate and for us to publish case details. Informed consent continued throughout the study via dialogue between researchers and participants.

### Experiment for creating gait animation

We recruited seven professional runway models (42.4 ± 7.0 years; 170.6 ± 3.7 cm; 55.6 ± 3.4 kg) and 10 non-models (34.0 ± 7.2 years; 162.0 ± 5.4 cm; 54.7 ± 7.7 kg). Our reasons for recruiting women from the general population (non-models) as well as professional fashion models and why we did not compare these groups were as follows: the study constructed models explaining female gait attractiveness and femininity. For this purpose, it was necessary to acquire data with a wide distribution. Therefore, we additionally acquired data from fashion models. As the study was not concerned with group differences in gait attractiveness, we did not conduct between-group comparisons. This is supported by our previous study that demonstrated common strategies to express gait attractiveness for models and non-models, indicating that it was possible for both groups to be evaluated with the same criteria for attractiveness and femininity^31^. However, the heights and ages of the groups varied considerably, potentially polarizing the data. Future studies are needed that can eliminate the effects for more equally matched age distributions by recruiting participants with a wider range in age and height.

**Figure 1:**
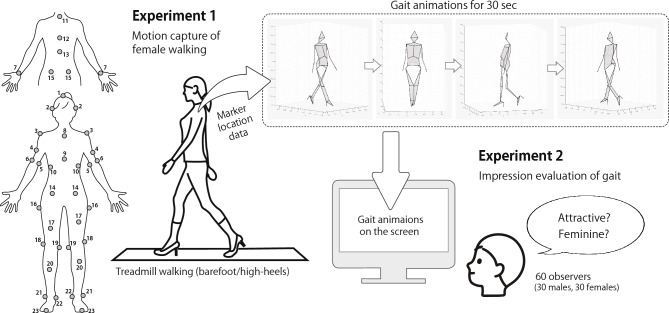
Schematic diagram of experiments 1 and 2 in a study of the factors associated with gait attractiveness. The attachment positions of reflective markers were 1. the top of the head; 2. the ear; 3. the acromion; 4. the upper arm; 5. the humerus-medial epicondyle (*elbow-in*); 6. the humerus-lateral epicondyle (*elbow-out*); 7. the wrist, 8. the upper margin of the sternum (*STER*); 9. the sternum-xiphoid process; 10. the lowest edge of the ribs; 11. the c7 vertebra; 12. the t8 vertebra; 13. the t12 vertebra; 14. the anterior superior iliac spine (*ASIS*); 15. the posterior superior iliac spine (*PSIS*); 16. the greater trochanter; 17. the femur; 18. the lateral knee joint space (*knee-out*); 19. the medial knee joint space (*knee-in*); 20. the shank; 21. the malleolus lateralis (*ankle-out*); 22. the malleolus medialis (*ankle-in*); 23. the toe and calcaneus (not shown).

The participants walked on a treadmill in high heels or barefoot (two trials for each condition) at a speed of 1.0 m/s. They were instructed to walk as usual, while thinking about things other than their own walking motion such as food and hobbies. We used a three-dimensional (3D) optical motion capture system (OptiTrack V100—R2; NaturalPoint, Corvallis, OR) composed of 12 infrared cameras with a sampling frequency of 100 Hz to obtain the following feature points’ positions of each participant’s body (42 markers in total; Fig. 1): the top of the head, ear, acromion, upper arm, humerus-medial epicondyle (*elbow-in*), humerus-lateral epicondyle (*elbow-out*), wrist, upper margin of the sternum (*STER*), sternum-xiphoid process, lowest edge of rib, C7 vertebra, T8 vertebra, T12 vertebra, anterior superior iliac spine (*ASIS*), posterior superior iliac spine (*PSIS*), greater trochanter, femur, lateral knee joint space (*knee-out*), medial knee joint space (*knee-in*), shank, malleolus lateralis (*ankle-out*), malleolus medialis (*ankle-in*), toe, and calcaneus. The *elbow-in*, *knee-in*, and *ankle-in* markers were removed during walking trials because they interfered with natural walking. Their coordinates were subsequently estimated in an offline analysis using three-point interpolation^51^.

The joint centers of the ankle, knee, and elbow were calculated as the midpoints between the lateral and medial markers for each joint. Hip coordinates were calculated using the marker location data of the greater trochanter and *ASIS*^52^. The trunk was represented as a double-link rigid body with lumbosacral, thoracolumbar, and neck joints, and the joint centers were calculated using the *ASIS* and *PSIS* (for the lumbosacral joint); the *STER*, C7, T8, T12, and rib (for the thoracolumbar joint); the *STER*, C7, and rib (for the neck); and the STER, C7, and acromion (for the shoulder)^53^. Then, time series data for joint center coordinates were then passed through a fourth-order Butterworth low-pass filter (cut-off frequency 6 Hz), using the *filtfilt* function in the MATLAB signal processing toolbox. Additionally, we used these filtered signals for three-dimensional calculations of the joint angles for the neck, shoulder, thoracolumbar, and knee. These were used in correlation analysis (Section "Correlation analysis of gait parameters and impression evaluation scores"). We created a 30-s gait animation using filtered joint center coordinates. During these 30 s, we rotated the animation’s viewpoint at a constant speed from the walker’s front right to back left (Videos 1 and 2 are sample animations from data of non-models barefoot and runway models wearing high heels, respectively). Animations numbered 68 (17 participants, two footwear conditions, and two trials for each). In addition, we calculated 3D joint angles for the ankle, knee, hip, lumbosacral joint, thoracolumbar joint, neck, shoulder, and elbow. These joint angle data were used to investigate motion factors that affect observers’ judgment of attractiveness and femininity (see Section "Correlation analysis of gait parameters and impression evaluation scores"). All signal processing was conducted using MATLAB R2021a.

### Experiments for impression evaluation

Thirty women (38.5 ± 13.3 years) and 30 men (40.7 ± 10.6 years) participated in evaluation of gait attractiveness and femininity. Participants viewed the 30-s walking animations presented on a standard computer monitor (EIZO FlexScan EV2480). To avoid participants’ evaluation of gait attractiveness and femininity based solely on early attentional acquisition in the first second^54^, we instructed participants to continue watching until the video finished playing. The presentation order was randomized, and participants were asked to keep their eyes on the animation while it was moving. Within 30 s after each animation stopped, participants judged attractiveness (“the walk is very attractive”) and femininity ("the walk is very feminine”) on a 7-point Likert scale with the following items: strongly disagree, disagree, slightly disagree, neither disagree nor agree, slightly agree, agree, strongly agree. This format is simple and easy for participants to understand, has sufficient measurement precision, and is presented with a single direction preference for positively worded items, which is essential for a valid Likert-scale^55^. Each item was scored from 1 for “strongly disagree” to 7 for “strongly agree.” Previous studies have used similar Likert scale questionnaires to evaluate the attractiveness and femininity/masculinity of the movements made by the body^7,25,27,48^. We provided participants no information about walkers (e.g., age, sex, occupation). Participants took a break after every 17 animation evaluations.

### Correlation analysis of gait parameters and impression evaluation scores

To obtain variables that explain attractiveness and femininity scores (A-score and F-score, respectively), we analyzed correlations between each score and the walkers’ physique (height, weight, BMI, and WHR), gait silhouette, and health factors under the barefoot and high-heel conditions. Since observers were not informed of walkers’ attributes (i.e., runway model or non-model), data from both groups were pooled. We calculated WHR using the horizontal difference between the left and right markers of the rib and greater trochanter as follows:

$${d}_{rib}=\sqrt{{\left({x}_{rib\_R}-{x}_{rib\_L}\right)}^{2}+{\left({y}_{rib\_R}-{y}_{rib\_L}\right)}^{2}},$$

$${d}_{tro}=\sqrt{{\left({x}_{tro\_R}-{x}_{tro\_L}\right)}^{2}+{\left({y}_{tro\_R}-{y}_{tro\_L}\right)}^{2}},$$

$$WHR={d}_{rib}/{d}_{tro},$$

Where *d*_*rib*_ and *d*_*tro*_ were the horizontal distances between the left and right ribs and the greater trochanters, respectively.

Silhouette elements consisted of the trunk silhouette (Silhouette-T), which included lumbar curvature and upper-arm backward swing, and the head silhouette (Silhouette-H), which included the head’s forward tilt and horizontal shaking. Based on our previous research on calculating the silhouette parameters that contributes to the expression of gait attractiveness^31^, both elements were calculated as follows: first, we obtained time series data for the upper body’s (*F*_*i*_) flexibility and limb and head alignment in a forward–backward direction (*A*_*i*_) according to the following equations:

$${F}_{i}=\left|{\theta }_{i}-{\overline{\theta }}_{i}\right|$$

where ${\uptheta }_{\mathrm{I}}$ represents the joint angle at the thoracolumbar and neck joints and where ${\overline{\theta }}_{i}$ is the mean joint angle in the sagittal and horizontal planes. ${F}_{i}$ of the null represents the joint’s complete immobility.

$${A}_{i}={r}_{i}\cdot \mathrm{sin}{\theta }_{i}$$

where ${\uptheta }_{\mathrm{i}}$ represents the joint angle at the neck, shoulder, and knee in the sagittal plane and where ${\mathrm{r}}_{\mathrm{i}}$ is the segment length of the head, upper arm, and shank. ${\mathrm{A}}_{\mathrm{knee}}$ was used for calculation of the knee extension parameter among health factors (see the next paragraph). ${\mathrm{A}}_{\mathrm{i}}$ of positive and negative directions represent backward and forward deviation of head and limb alignment. The lumbar curvature that constitutes Silhouette-T was calculated as the average value of the entire gait cycle period of ${F}_{thoracolumbar}$, and the backward upper-arm swing as the maximum value of the entire gait cycle period of ${A}_{shoulder}$ (i.e., the upper arm’s posterior position). In addition, the head’s forward tilt and horizontal shaking that made up Silhouette-H were average values of the entire gait cycle period of $(-1)\cdot {A}_{neck}$, and the average of ${F}_{neck,}$ the periods before and after foot contact (i.e., 0–10, 40–60, 90–100% of gait cycle).

Considering findings from previous research that states that “the animals’ standard of beauty, including that of human beings, is associated with psychological processes that detect health-related features”^1–6^, the gait parameters related to walkers’ health and aging may be linked to gait attractiveness. Thus, we composed health factors of the following health-related gait parameters: stride CV, cadence, clearance, symmetry, knee extension, and toe-off angle at push-off phase^35–40^. Stride CV is the coefficient of variation of time required for one gait cycle, and cadence was calculated as the number of steps per minute. To calculate the clearance, we first detected the minimum vertical distance from the floor to the toe during the swing phase for each leg and normalized it by each walker’s height. Then, clearance was defined as the average value of the normalized left and right distances. Symmetry was expressed as the ratio of the left and right average swing times, and the lesser of those swing times was used as the denominator; that is, the greater the symmetry value, the more asymmetric. To calculate the parameter of knee extension during the push-off phase, mean $(-1)\cdot {A}_{knee}$ values during the push-off phase of each leg (left leg: 0–10% gait cycle; right leg: 50–60% gait cycle) were summed (i.e., average ${A}_{knee\_L}+{A}_{knee\_R}$ during the push-off phase). Furthermore, to calculate the toe-off angle at push-off, the angle formed by the line segment connecting the toe and heel, and the horizontal plane at toe-off was averaged over all steps for each leg; then, we averaged left and right toe-off angles.

Finally, we performed correlation analysis between the parameters above and impression evaluation scores (A- and F- scores) using the Pearson’s correlation coefficient test. Parameters with moderate or higher correlation (i.e., correlation coefficient |r|> 0.3 and *p* ≤ 0.05) were used as observed variables in subsequent structural equation modeling.

### Structural equation modeling (SEM)

To construct a model that explains the attractiveness and femininity of women’s gait, we performed SEM^56^ incorporating walkers’ physique and gait parameters that correlated with the A-score or the F-score as predictor variables. Unlike linear regression models that assume predictor variables are fixed or measured without error^57^, SEM can combine measurement models with structural (i.e., regression) models into one overarching model that optimally handles measurement error in predictor variables. In this study, we constructed a hypothesized model to be tested by using parameters correlated with the A-score and/or F-score as predictor variables with latent variables of physique, gait silhouette, and health factors. Runway model and non-model data were pooled to test the hypothesized model for each footwear condition. Data samples numbered 2037 and 2038 for the barefoot and high-heel conditions, respectively (17 walkers × 2 trials × 60 observations).

We performed SEM using the SPSS Amos software (IBM SPSS 29.0, Amos Version 29) with a visual and intuitive interface. Model evaluation was performed using the chi-square test statistic and the model’s overall fit indices provided by the SPSS Amos software. Ideally, for a model that fits the data, the χ2 would not be significant (p > 0.05). However, because SEM is based on covariances, chi-square test of fit is sensitive to sample size: as sample size increases with the constant degrees of freedom, the χ2 value increases, thus resulting in small *p* value. Such dependence on sample size leads to the problem that plausible models might be rejected based on a significant χ2 statistic even though the discrepancy between the sample and the model-implied covariance matrix is irrelevant. In contrast, as sample size decreases, the χ2 value decreases as well and the model test may indicate nonsignificant probability levels even though the discrepancy between the sample and the model-implied covariance matrix is considerable. Therefore, not too much emphasis should be placed on the significance of the χ2 statistic^58^.

We also evaluated the model’s fit according to four popular fit indices: the goodness of fit index (GFI;^59,60^), the adjusted goodness of fit index (AGFI;^59^), the comparative fit index (CFI;^61^), and the root mean square error of approximation (RMSEA;^62,63^). The GFI ranges from zero to one, with higher values indicating better fit, and the usual rule is that GFI > 0.95 indicates good fit relative to the baseline model, while GFI > 0.90 is usually interpreted as acceptable fit^64,65^. AGFI also typically ranges from zero to one, with greater values indicating better fit; AGFI > 0.90 indicates good fit relative to the baseline model, while AGFI > 0.85 could be considered acceptable. AGFI is usually smaller than GFI, and AGFI approaches GFI as the target model’s degrees of freedom approach those of the null model. Hu and Bentler^66,67^ suggested also that GFI and AGFI are not independent of sample size and that both indices decrease with increasing model complexity, especially for smaller sample sizes^68^. In addition, CFI > 0.95 is often indicative of good fitting models^67^. RMSEA, which estimates a model’s lack of fit and is a measure of non-centrality relative to sample size and degree of freedom, indicates a close-fitting model with values of 0.06 or less^67^, while values greater than 0.10 indicate a poor-fitting model^63^. Details of these fit measures’ definitions and calculations can be found in Schermelleh-Engel et al.^58^.

## Results

### Correlation analysis of gait parameters and impression evaluation scores

To obtain predictor variables of gait attractiveness and femininity, we analyzed correlations between each score and the walker’s physique, gait silhouette, and health factors under barefoot and high-heel conditions. Tables 1, 2, 3 show results of correlation analysis for physique, gait silhouette, and health factors, respectively. Among the height, weight, BMI, and WHR constituting physique factors, only BMI correlated with both A- and F-scores for both footwear conditions (Table 1), which indicated that the lower the BMI, the more attractive and feminine. In addition, Lumbar curvature showed positive correlation with both A- and F-scores only for the barefoot condition (Table 2). Backward arm swing and forward head tilt positively correlated with both A- and F-scores in both footwear conditions (Table 2). These results could be interpreted as follows; swinging your arms behind your body, tilting your head downwards, and curving your back when not wearing high heels could make you attractive and feminine. In contrast, the horizontal head shake during periods before and after foot contact correlated negatively with both A- and F-scores only for the high-heel condition (Table 2), indicating that both A- and F-scores are higher when head -shaking motion in the horizontal plane is not linked to the shoulders’ horizontal rotation and the head is always facing forward.

**Table 1:** Pearson’s correlation coefficient for physique factors.

		Barefoot		Heels	
		A-score	F-score	A-score	F-score
Height	r	.258	.242	.296	.317
	*p* value	.141	.167	.089	.068
Weight	r	− .328	− .339	− .269	− .306
	*p* value	.059	.050	.123	.078
BMI	r	**− .566**	**− .567**	**− .528**	**− .585**
	*p* value	**< .001**	**< .001**	**.001**	**< .001**
WHR	r	− .151	− .211	− .147	− .208
	*p* value	.394	.230	.407	.238

Significant values are in bold.

**Table 2:** Pearson’s correlation coefficient for gait silhouette factors.

		Barefoot		Heels	
		A-score	F-score	A-score	F-score
Lumbar curvature	r	**.420**	**.387**	.032	.085
	*p* value	**.013**	**.024**	.858	.632
Backward arm swing	r	**.717**	**.588**	**.660**	**.643**
	*p* value	**< .001**	**< .001**	**< .001**	**< .001**
Forward head tilt	r	**.652**	**.718**	**.675**	**.727**
	*p* value	**< .001**	**< .001**	**< .001**	**< .001**
Horizontal head shake	r	− .277	− .245	**− .438**	**− .451**
	*p* value	.113	.163	**.010**	**.007**

Significant values are in bold.

**Table 3:** Pearson’s correlation coefficient for health factors.

		Barefoot		Heel	
		A-score	F-score	A-score	F-score
Stride CV	r	− .322	− .292	− .292	− .290
	*p* value	.063	.093	.094	.096
Cadence	r	**− .442**	**− .433**	**− .383**	**− .454**
	*p* value	**.009**	**.011**	**.025**	**.007**
Clearance	r	− .138	− .030	**− .364**	− .308
	*p* value	.438	.862	**.034**	.076
Symmetry	r	.017	− .049	− .132	− .166
	*p* value	.925	.783	.457	.348
Knee extension	r	**.339**	**.407**	**.533**	**.525**
	*p* value	**.050**	**.017**	**.001**	**.001**
Toe-off angle	r	**.349**	**.507**	.302	.294
	*p* value	**.043**	**.002**	.083	.091

Significant values are in bold.

Cadence correlated negatively with both A- and F-scores for both footwear conditions (Table 3), indicating that walking with long stride time is more attractive and feminine. In addition, knee extension parameter and toe-off angle at push-off correlated positively with both scores for both conditions and only for barefoot condition, respectively. These results show that it is more attractive and feminine to extend the knee and raise the heel when the rear leg push-off the ground. Clearance correlated negatively with only the A-score (although it also tended to correlate negatively with the F-score) for only the high-heel condition, indicating that closer foot-to-floor distance during swing phase may lead to attractiveness evaluation.

### Modeling gait attractiveness and femininity with SEM

Based on correlation analysis results (Tables 1, 2, 3), we determined the predictor variables of hypothesized models for each footwear condition as follows: for a model of the barefoot condition, BMI as a physique factor; lumbar curvature and backward arm swing as gait silhouette factors (Silhouette-T); forward head tilt as the other gait silhouette factor (Silhouette-H); and cadence, knee extension, and toe-off angle as health factors. For a model of the high-heel condition, we adopted BMI as the physique factor; backward arm swing as the gait silhouette factor (Silhouette-T); forward head tilt and horizontal head shake as the other gait silhouette factor (Silhouette-H); and cadence and knee extension as health factors. Because correlation was relatively weak between clearance and A-score for the high-heel condition, we did not include clearance as a predictor variable.

**Figure 2:**
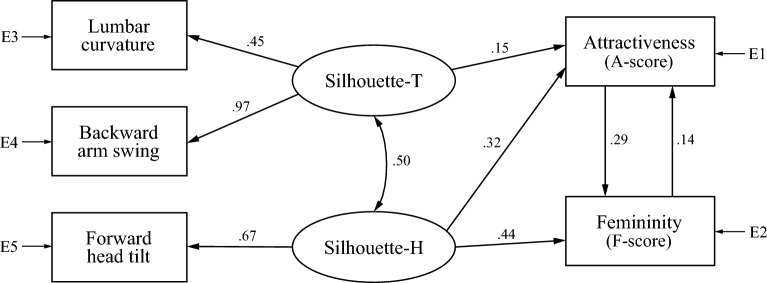
Model B1, composed of two gait silhouette factors (Silhouette-T and Silhouette-H), for gait attractiveness and femininity in the barefoot condition. E1 to E5 stand for measurement errors.

**Figure 3:**
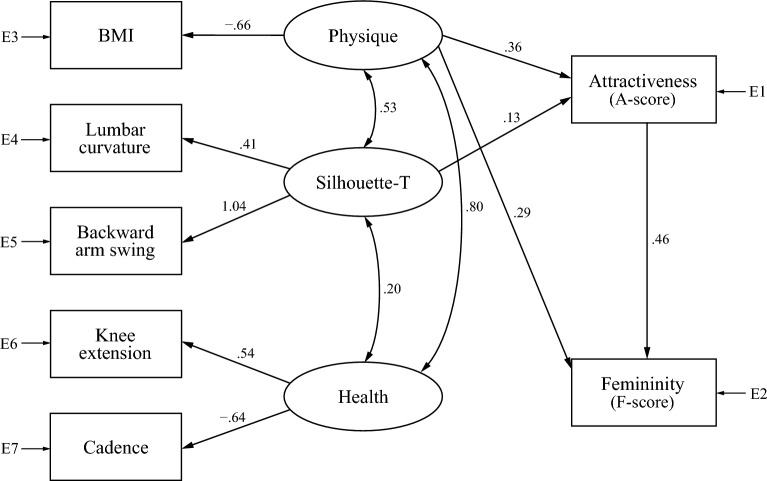
Model B2, composed of physique, Silhouette-T, and health factors, for gait attractiveness and femininity in the barefoot condition. E1 to E7 stand for measurement errors.

We found two models to explain A- and F-scores for the barefoot condition, one composed of both silhouette factors (Model B1; Fig. 2) and the other of physique, Silhouette-T, and health factors (Model B2; Fig. 3). In the figures, latent variables are represented with circles, and measured variables with squares. A line with an arrow indicates a direct relationship between variables with the estimated standardized coefficients on every path. A line with a two-headed arrow indicates a relationship, that is, a covariance, between the two variables with no implied direction of effect. E1 to E7 in the Figures stand for measurement errors. We removed paths with no direct relationship having an absolute standardized coefficient value less than 0.1. We excluded the toe-off angle variable in Models B1 and B2 because the model did not converge when the health factor toe-off angle was added as a predictor.

In Model B1 (Fig. 2), signs of all standardized estimates were consistent with correlation analysis results. Chi-square test results and model fit evaluation were as follows: χ2 = 34.63 with 2 *dfs*, p < 0.001; GFI = 0.993, AGFI = 0.949, CFI = 0.965, and RMSEA = 0.090. Although chi-square was significant, GFI, AGFI, and CFI exceeded cut-off values of 0.95, 0.90, and 0.95, respectively, and RMSEA did not exceed the cut-off value of 0.10 for the poor-fitting model. From these results, we can conclude that Model B1 generally fits well. There was a moderate mutual relationship between both silhouette factors (standardized estimate = 0.50), and the Silhouette-T factor had a direct effect only on attractiveness. Additionally, in Model B1, gait attractiveness and femininity had a positive mutual relationship.

In Model B2 (Fig. 3), none of the standardized estimates’ signs disagreed with the correlation analysis results. Chi-square test results and model fit evaluation were as follows: χ2 = 80.30 with 9 *dfs*, *p* < 0.001 GFI = 0.989, AGFI = 0.965, CFI = 0.940, and RMSEA = 0.062. Although chi-square was significant, GFI and AGFI exceeded cut-off values of 0.95 and 0.90, respectively. In addition, CFI was close to its cut-off value of 0.95, which could be considered acceptable. Although RMSEA was outside the cut-off value of 0.06 for a close-fitting model, it did not exceed the cut-off value of 0.10 for the poor-fitting model. Therefore, we can conclude that Model B2 also generally fits well. There was a mutual relationship between all latent variables, especially between physique and health factors (standardized estimate = 0.80). Although there were direct paths from the physique factor to both attractiveness and femininity, there were no paths from the health factor, indicating that health factors indirectly affect attractiveness and femininity through physique and Silhouette-T factors. Even in Model B2, the Silhouette-T factor had a direct effect only on attractiveness. Additionally, unlike Model B1, this model showed no direct path from femininity to attractiveness. We attempted to combine Models B1 and B2 to construct one model for barefoot walking, but the model did not converge, likely due to a discrepancy between the two models. Model B1 has bi-directional paths between attractiveness and femininity, whereas Model B2 draws a path only from attractiveness to femininity.

**Figure 4:**
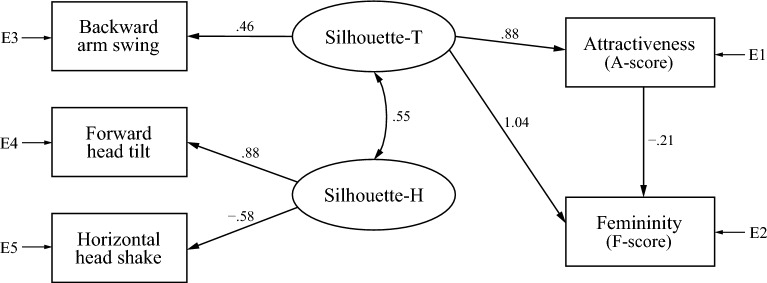
Model H1, composed of two gait silhouette factors (Silhouette-T and Silhouette-H), for gait attractiveness and femininity in the heel condition. E1 to E5 stand for measurement errors.

**Figure 5:**
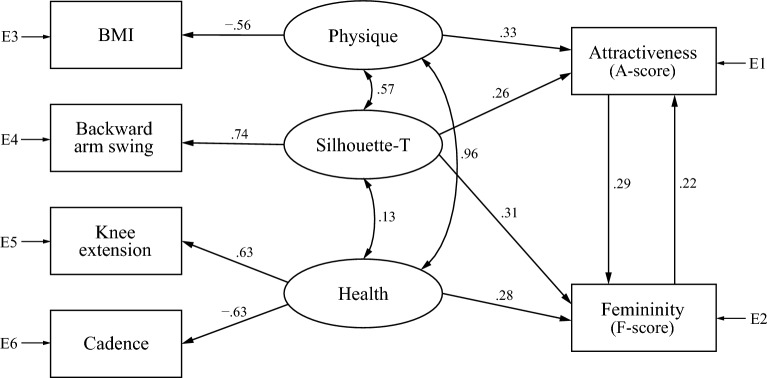
Model H2, composed of physique, Silhouette-T, and health factors, for gait attractiveness and femininity in the heel condition. E1 to E6 stand for measurement errors.

We also found two models to explain the A-score and F-score for the high-heel condition, one composed of both silhouette factors (Model H1; Fig. 4), and the other of physique, Silhouette-T, and health factors (Model H2; Fig. 5). Additionally, in these models, we removed paths lacking a direct relationship that had an absolute standardized coefficient value of less than 0.1. In the high-heel condition, we have observed a negative correlation between clearance and A-score (Table 1). Thus, we initially performed SEM by adding clearance to Model H2 as an explanatory variable; however, the model did not converge. Therefore, we have excluded clearance from Model H2 (Fig. 5).

In Model H1 (Fig. 4), all standardized estimates’ signs were consistent with correlation analysis results. Chi-square test results and model fit evaluation were as follows: χ2 = 32.43 with 3 *dfs*, p < 0.001; GFI = 0.994, AGFI = 0.968, CFI = 0.971, and RMSEA = 0.069. Although chi-square tests were significant, GFI, AGFI, and CFI exceeded cut-off values of 0.95, 0.90, and 0.95, respectively. RMSEA did not exceed the poor-fitting model cut-off of 0.10, and it nearly approached the cut-off of 0.06 for a close-fitting model. From these results, we concluded that Model H1 generally fits well. Similar to Model B1, Model H1 had a moderate mutual relationship between both silhouette factors (standardized estimate = 0.55). The Silhouette-T factor had a direct effect on both attractiveness and femininity. Interestingly, the standardized estimate of the path from Silhouette-T to femininity was greater than 1, and the direct effect from attractiveness to femininity was negative (standardized estimate =  − 0.21). This suggests that, in high-heel walking, the backward arm swing of Silhouette-T contributes to both attractiveness and femininity, but the direct relationship between attractiveness and femininity is that the more attractive the gait, the less feminine it is.

Figure 5 shows Model H2, the other model for gait attractiveness and femininity of high-heel walking. All standardized estimates’ signs were consistent with correlation analysis results. Chi-square test results and model fit evaluation were as follows: χ2 = 54.71 with 3 *dfs*, *p* < 0.001; GFI = 0.991, AGFI = 0.937, CFI = 0.951, and RMSEA = 0.092. Although chi-square tests were significant, GFI, AGFI, and CFI exceeded cut-off values of 0.95, 0.90, and 0.95, respectively. In addition, RMSEA did not exceed the cut-off value of 0.10 for the poor-fitting model. Thus, we concluded that Model H2 also generally fits well. Similar to Model B2, Model H2 had mutual relationships between all latent variables, especially between physique and health factors (standardized estimate = 0.96). The Silhouette-T factor had a direct effect on both attractiveness and femininity. However, physique and health factors had direct effects only on attractiveness and femininity, respectively. In this model, similarly to Model B1, gait attractiveness and femininity had a positive mutual relationship. We also attempted to combine Models H1 and H2 into one model for high-heel walking, but they did not converge, probably because of a discrepancy between the two; Model H2 had bi-directional paths between attractiveness and femininity, whereas Model H1 drew a path only from attractiveness to femininity, and its effect was negative.

## Discussion

Regarding Hypothesis 1, the hypothesis was partially supported. Only BMI was correlated with A- and F-scores in both footwear conditions (Table 1), indicating that the lower the BMI, the more attractive and feminine the walker. This is consistent with previous findings^21^. Several studies have reported WHR as a criterion for evaluating female attractiveness^6,12^; however, our results found no correlation between walkers’ WHR and observers’ impression scores. This may be because the WHR data had insufficient variability to produce a corresponding variability in impression ratings. The WHR of our walking animations was 0.69 ± 0.034 (ranging from 0.627 to 0.738, below the < 0.80^69^ specified as a good health level), while in previous studies, they have achieved a wide range of WHR values (0.7–1.0 in^6^; 0.5–0.9 in^12^) by artificially modifying stimuli. Thus, our results suggest that small differences in WHR do not affect gait attractiveness and femininity. Future research using gait stimuli with larger WHR (about 0.8–0.9) may find WHR to be related to impression evaluation.

Hypothesis 2 was supported; lumbar curvature was positively correlated with A- and F-scores for the barefoot condition. This finding is in line with previous studies showing that women with a more curved lumbar spine were evaluated as more attractive^7^. Backward arm swing and forward head tilt were positively correlated with A- and F-scores for both footwear conditions (Table 2). This suggests that when not wearing high heels, swinging the arms behind the body, tilting the head downward, and curving the back may affect attractiveness and femininity. In addition, horizontal head shaking before and after foot contact was negatively correlated with A- and F-scores for the high-heel condition, indicating that A- and F-scores are higher when head -shaking motion in the horizontal plane is not linked to the horizontal rotation of the shoulders and when the head faced forward. To the best of our knowledge, no research has examined the relationship between head silhouette and bodily attractiveness. Given that static rather than dynamic poses are preferred for female bodies^48^, a forward head tilt, which is related to the expression of sadness^70,71^, and less horizontal head shaking could be considered to enhance the static impression of gait, leading to the attractiveness evaluation.

Regarding Hypothesis 3, the hypothesis was partially supported. Among health factor candidates, cadence, knee extension, toe-off angle, and clearance were correlated with A- and F-scores (Table 3), indicating that walking with long stride time and extending the knee and raise the heel at the push-off is more attractive and feminine. The results of correlation analysis on knee extension and toe-off angle aligned with evolutionary psychology’s view that animals’ standard of beauty is associated with psychological processes detecting health-related features^1–6^. Lower cadence reflects a reduced ability to modulate gait cycle duration and is linked to age-related disease (such as knee osteoarthritis)^40^. However, in our analyses, low cadence was associated with attractiveness and femininity. In addition, clearance was negatively correlated with the A-score for the high-heel condition, indicating that closer foot-to-floor distance, which increase the risk of falls^36^, is associated with attractiveness evaluation. These results partially contradict our hypothesis as, contrary to expectations, small cadence and clearance were correlated with gait attractiveness. Thus, gait attractiveness is partially independent of the health and youth of walkers. However, in the subsequent SEM, clearance and toe-off angle parameters did not fit Models B2 and H2 (Figs. 3 and 5, respectively); that is, the models did not converge with the addition of these parameters. There are two possible reasons for this result. First, the correlation between these parameters and impression evaluation is weak, and second, such correlation could be a byproduct of other gait features associated with attractiveness. In contrast, Models B2 and H2 incorporated knee extension and cadence, showing for the first time that gait attractiveness and gait parameters could be related.

Interestingly, our analysis found correlations between lumbar curvature and toe-off angle and A- and F-scores but only in the barefoot condition (Tables 2 and 3). There are three possible reasons for this result. First, these parameters are not related to the attractiveness and femininity of high-heel walking; second, wearing high heels passively raises the heel and lumbar curvature in every walker, which reduces inter-individual variation in these parameters and makes it impossible to obtain correlations with impression evaluation scores; or third, the toe-off angle and lumbar curvature are sufficient for obtaining high A- and F- scores by wearing high heels and are not related to the detection of differences in impression evaluations. In other words, these parameters are no longer used to determine gait attractiveness and femininity. To explore this, we performed statistical comparisons of the lumbar curvature and toe-off angle between the footwear conditions. We found that the toe-off angle was significantly larger in the high-heel condition (37.16 ± 2.77 and 53.98 ± 7.75 for barefoot and high-heel conditions, respectively; *t*(66) = 11.91; *p* < 0.001; Cohen’s *d* = 2.86). The coefficient of variation was 0.0745 and 0.144 for barefoot and high-heel conditions, respectively; thus, the variation was larger in the high-heel condition. This supports the third possibility regarding the toe-off angle. On the other hand, there was no significant difference in lumbar curvature between the two footwear conditions (1.44 ± 0.46 and 1.56 ± 0.56 for barefoot and high-heel conditions, respectively; *t*(66) = 0.98; *p* = 0.32; Cohen’s *d* = 0.24). This supports the first possibility regarding lumbar curvature. In addition, regarding the differences in our modeling of barefoot and high-heel walking, we found that the horizontal head-shake parameter was negatively correlated with A- and F-scores in the high-heel condition (Table 2). Unlike parameters that may be intentional motion manipulations by the walker, the horizontal head shake is likely an effect of the changes in horizontal shoulder rotation associated with changes in the walker’s body alignment caused by wearing high heels. As far as we know, no research has been conducted on the relationship between horizontal head movement and impression evaluation of walking; thus, interpersonal cognition brought about by head movements would require further investigation.

**Figure 6:**
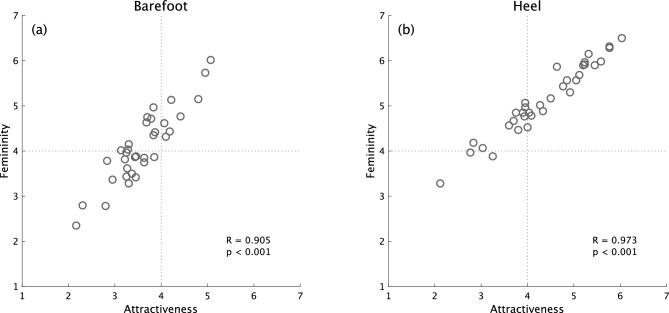
Scatter plots of attractiveness and femininity scores for (**a**) barefoot and (**b**) high-heel walking. Each plot represents 60 observers’ average score for each gait animation.

Our correlation analysis found no difference between the A-score and the F-scores of correlated parameters except for those regarding the clearance parameter. We found a strong positive correlation between attractiveness and femininity scores: r = 0.905 and *p* < 0.001 for the barefoot condition, and r = 0.973 and *p* < 0.001 for the high-heel condition (Fig. 6). Thus, gait attractiveness and femininity appeared equivalent at the correlation analysis level. Therefore, in subsequent SEM, we investigated the relationship between gait attractiveness and femininity. The final goal of this study was to construct models of female gait attractiveness with gait kinematics and physical characteristics as explanatory variables. We created four latent variables based on the parameters’ quality: physique, trunk silhouette, head silhouette, and health factors. Two models were constructed by SEM for barefoot and high-heel walking, respectively: one consisted of trunk and head silhouette factors (Models B1 and H1), and the other of physique, trunk silhouette, and health factors (Models B2 and H2). These results suggest that the norm for women’s gait attractiveness and femininity might consist of at least these four factors. None of our constructed models had sufficiently small chi-squared values for *p* values to exceed 0.05, possibly due to the large sample size because “with increasing sample size and a constant number of degrees of freedom, the chi-squared value increases. This leads to the problem that plausible models might be rejected based on a significant chi-squared statistic even though the discrepancy between the sample and the model-implied covariance matrix is actually irrelevant”^59^.

The study’s two models constructed by SEM shared the same latent variables between barefoot and high-heel conditions: one is a model composed of trunk and head silhouette, and the other of physique, trunk silhouette, and health factors. This suggests that evaluation criteria for gait attractiveness and femininity are consistent, regardless of footwear. However, the biggest difference in SEM results between the barefoot and high-heel conditions was that attractiveness affected perceptions of femininity positively in Model B2 and negatively in Model H1. Although attractiveness and femininity scores were highly correlated (Fig. 6), the Model H1 suggested a negative causal relationship in the direct relationship between gait attractiveness and femininity. Furthermore, although the two models for barefoot (Models B1 and B2) and high-heel (Models H1 and H2) walking shared the trunk silhouette factor, combining the two models was not possible. This suggests that two different evaluation norms might function for gait attractiveness and that the trunk silhouette might have two different informational values for attractiveness perception. These models’ main expressed difference was in the relationship between attractiveness and femininity. For example, in Models B1 and H2, gait attractiveness caused femininity and vice versa, while in Model B2, attractiveness mediated gait kinematics and femininity evaluation. Interestingly, in contrast to Model B2, attractiveness perception in Model H1 produced negative femininity evaluation (i.e., attractive gait was evaluated as masculine). This suggests three types of relationships between perceived attractiveness and femininity: (1) an equivalence relationship; (2) a relationship in which attractiveness induces femininity perception; and (3) a relationship in which attractiveness negatively influences femininity perception. The equivalence relationship is considered the model behind the strong link between perceptual dimensions of femininity and attractiveness, reported by Sadr et al.^26^, in which artificially feminized walkers were rated much more attractive than averaged female walking. The second and third relationships, however, appear to take the opposite direction of the causal relationship between attractiveness and femininity compared to the previous study, where less dynamism in body pose led to the observer’s feelings of pleasantness through femininity^48^. Because Sadr et al.^26^ did not verify that pleasantness perception causes femininity, an equivalent relationship between liking and femininity might have existed in their experimental data. Another possibility is that their study evaluated body poses using still images, whereas our study evaluated attractiveness by observing dynamic gait motion. In other words, since brain regions involved in perceiving the body’s emotional state (e.g., the STS region, which plays an important role in social information perception;^72,73^) partially differ depending on whether presented stimuli are static or dynamic^74,75^, this study’s evaluation of the impression of gait animation was performed using a different pathway from that of static stimuli.

This study has three main limitations. First, the walkers’ heights and ages differed considerably between the groups, potentially polarizing the data. Due to the nature of the fashion model profession, it is difficult to recruit fashion models of similar height to non-models; however, future studies should recruit participants with a wider range of age participants for more equally matched distributions between the groups. Second, our data was obtained from Japanese participants; thus, the results are likely to be biased toward Japanese culture. The applicability of our findings to other locations and cultures requires further investigation. Third, our judgments of the attractiveness of a person when they are walking is influenced not only by their body movements but also by the context, situation, environment, and their clothing. Future research should clarify these diverse contributors to the attractiveness of body movements.

Through this research, we constructed models that connect the walker’s gait kinematics and the observer’s perception of attractiveness. Results suggested two models behind gait attractiveness in both barefoot and high-heel walking: one composed of trunk and head silhouette factors and the other of physique, trunk silhouette, and health-related gait factors. These models clarified the gait kinematics and physique factors that lead to gait attractiveness and femininity. Thus, we could conclude that the theoretical foundation of female gait attractiveness is based on the walkers’ physique (low BMI), a trunk silhouette that emphasizes the feminine form (lumbar curvature and backward arm swing), head silhouette (forward tilt and less horizontal shaking), and health- and age-related gait parameters such as cadence and knee extension. Since predicting the target variable from SEM-based models is possible^76^, these parameters may be used to predict gait attractiveness and femininity values. The study’s results have deepened our understanding of mechanisms underlying nonverbal interpersonal cognition through physical movement. In addition, business (e.g., fashion-related industries^77^) and education’s^78^ current expansion on the Metaverse is expected to increase demand for algorithms that artificially generate biological motions affecting interpersonal impressions and facilitating interpersonal communication. This study’s results can contribute to such industries’ development through the possibility of generating attractive gait motions.

### Supplementary Information

Supplementary Video 1.

Supplementary Video 2.

## Data Availability

The datasets used and/or analyzed in this study are available from the corresponding author on reasonable request.
